# Vitamin K Intake and Plasma Desphospho-Uncarboxylated Matrix Gla-Protein Levels in Kidney Transplant Recipients

**DOI:** 10.1371/journal.pone.0047991

**Published:** 2012-10-31

**Authors:** Paul Y. Boxma, Else van den Berg, Johanna M. Geleijnse, Gozewijn D. Laverman, Leon J. Schurgers, Cees Vermeer, Ido P. Kema, Frits A. Muskiet, Gerjan Navis, Stephan J. L. Bakker, Martin H. de Borst

**Affiliations:** 1 Department of Internal Medicine, Division of Nephrology, University Medical Center Groningen and University of Groningen, Groningen, The Netherlands; 2 Top Institute Food and Nutrition, Wageningen, The Netherlands; 3 Division of Human Nutrition, Wageningen University, Wageningen, The Netherlands; 4 Department of Internal Medicine, Division of Nephrology, ZGT Hospital Almelo, Almelo, The Netherlands; 5 Department of Biochemistry, Maastricht University, Maastricht, The Netherlands; 6 VitaK, Maastricht University, Maastricht, The Netherlands; 7 Department of Laboratory Medicine, University Medical Center Groningen and University of Groningen, Groningen, The Netherlands; Innsbruck Medical University, Austria

## Abstract

Vitamin K is essential for activation of γ-carboxyglutamate (Gla)-proteins including the vascular calcification inhibitor matrix Gla-protein (MGP). Insufficient vitamin K intake leads to production of uncarboxylated, mostly inactive proteins and contributes to an increased cardiovascular risk. In kidney transplant recipients, cardiovascular risk is high but vitamin K intake and status have not been defined. We investigated dietary vitamin K intake, vascular vitamin K status and its determinants in kidney transplant recipients. We estimated vitamin K intake in a cohort of kidney transplant recipients (n = 60) with stable renal function (creatinine clearance 61 [42–77] (median [interquartile range]) ml/min), who were 75 [35–188] months after transplantation, using three-day food records and food frequency questionnaires. Vascular vitamin K status was assessed by measuring plasma desphospho-uncarboxylated MGP (dp-ucMGP). Total vitamin K intake was below the recommended level in 50% of patients. Lower vitamin K intake was associated with less consumption of green vegetables (33 vs 40 g/d, p = 0.06) and increased dp-ucMGP levels (621 vs 852 pmol/L, p<0.05). Accordingly, dp-ucMGP levels were elevated (>500 pmol/L) in 80% of patients. Multivariate regression identified creatinine clearance, coumarin use, body mass index, high sensitivity-CRP and sodium excretion as independent determinants of dp-ucMGP levels. In a considerable part of the kidney transplant population, vitamin K intake is too low for maximal carboxylation of vascular MGP. The high dp-ucMGP levels may result in an increased risk for arterial calcification. Whether increasing vitamin K intake may have health benefits for kidney transplant recipients should be addressed by future studies.

## Introduction

Vitamin K deficiency is increasingly recognized as a risk factor for cardiovascular morbidity and mortality in renal patients [1], [2]. Vitamin K refers to a set of different fat-soluble vitamins occurring as phylloquinone (vitamin K1) or a series of vitamins commonly termed menaquinones (vitamin K2). The main sources of vitamin K1, the most prominent form of vitamin K in the Western diet, are green vegetables and dairy products. Vitamin K2 comes from fermented food such as cheese and curd [3], [4], and is mainly considered to be produced by bacterial flora in the intestinal tract. Both vitamers serve as cofactors for modifying glutamate into gamma-carboxylated glutamate (Gla) residues in biological active proteins, including matrix Gla protein (MGP) [5]. In addition, vitamin K2 is a membrane-bound electron carrier in mitochondria [6]. MGP, which is synthesized by vascular smooth muscle cells and chondrocytes, is an important inhibitor of vascular calcification [7]. Poor vitamin K status due to poor intake or the use of vitamin K antagonists results in high uncarboxylated MGP (ucMGP) levels and is associated with vascular calcification, both in populations with and without renal disease [7], [8]. The plasma desphospho-ucMGP (dp-ucMGP) fraction is considered a marker for vascular vitamin K status [9], [10]. The actual contribution of dietary vitamin K intake to the vascular vitamin K status is not yet known, supplementation with menaquinone-7 (one of the K2 vitamins) may reduce dp-ucMGP levels in hemodialysis patients [11].

It has recently been demonstrated that the majority of hemodialysis patients have vitamin K deficiency as reflected by high dp-ucMGP levels [1], as well as low vitamin K intake [12]. Their low vitamin K intake may derive from the dietary regimen generally prescribed to hemodialysis patients, which includes restriction of sodium and potassium intake. Therefore dialysis patients limit their intake of mainly green vegetables and cheeses, i.e food products that are rich in vitamin K1 and K2, respectively. Factors other than dietary intake such as compromised renal function may contribute to the vitamin K status as well [13]. The vitamin K insufficiency present in the majority of hemodialysis patients may contribute to their strongly increased risk for arterial calcification development [14]–[16].

Although cardiovascular morbidity and mortality after kidney transplantation are lower compared to any of the dialysis modalities, the risks are considerably higher than those in the general population [17]. Whether vitamin K insufficiency is also common in kidney transplant recipients is unknown, but several factors associated with reduced vitamin K status such as impaired renal function remain present in many patients after kidney transplantation, and thus could affect vitamin K status. A recent study documented that kidney transplant recipients consume less sodium and potassium than the general population [18], but their dietary vitamin K intake has not been documented.

The objective of this study was to determine the intake of vitamin K1, vitamin K2 and total vitamin K and vascular vitamin K status, by measuring desphospho-ucMGP (dp-ucMGP) levels, in kidney transplant recipients. Furthermore, we aimed to identify dietary factors that are associated with vitamin K status in this patient group.

## Materials and Methods

### Ethics statement

The study was approved by the medical ethics committee of the University of Groningen (METc 2008/186), and all participants provided written informed consent. All clinical investigations have been conducted according to the principles expressed in the Declaration of Helsinki.

### Study population

No formal power calculation was performed for this explorative observational study. Kidney transplant recipients attending the outpatient clinic of the University Medical Center Groningen were recruited. Patients with a minimum age of 18 years and able to return completed food records were eligible, irrespective of gender, the underlying primary renal disease, the presence of cardiovascular disease, diabetes mellitus, or other traditional cardiovascular risk factors. Exclusion criteria were known malignancy, abnormal liver function tests, history of gastrointestinal disease or metabolic disease, or active infection. In total, 60 patients with a functioning kidney graft for at least one year were enrolled.

Standard immunosuppressive therapy was as follows: from 1968 until 1989 prednisolone (10 mg/day) and azathioprine (100 mg/day). From January 1989 until February 1993 ciclosporin standard formulation (Sandimmune, Novartis; 10 mg/kg; trough levels of 175–200 mg/l in first 3 months, 150 mg/l between 3 and 12 months post-transplant and 100 mg/l thereafter) combined with prednisolone (starting with 20 mg/day, rapidly tapered to 10 mg/day). From March 1993 until May 1996 ciclosporin microemulsion (Neoral, Novartis Pharma b.v., Arnhem, The Netherlands; 10 mg/kg; trough levels idem) and prednisolone. From May 1996 to date mycophenolate mofetil (MMF) (Cellcept, Roche b.v., Woerden, The Netherlands; 2 g/day) was added. Current medication was extracted from the medical records.

### Vitamin K intake

Dietary intake was assessed using a dietary diary that was kept during three consecutive days in advance of the patients' visit to the outpatient clinic. All patients adhered to their normal dietary habits. A trained researcher checked whether diaries were filled out properly, and if necessary additional information was obtained about unusual or missing reports. Intake was recorded in household measurements and standard portion sizes. For calculations of the intakes of total energy and nutrients, we used the Food Calculation System (BAS nutrition software 2004, Arnhem, The Netherlands) in which Dutch food composition database NEVO 2006 was included [19]. Concentrations of vitamin K1 and K2 (MK-4 through MK-10) of 260 foods have been added to the NEVO (2006) food database, as described previously [15]. Dietary intake of total vitamin K, vitamin K1 and vitamin K2 from three consecutive days were averaged and used for analysis.

The U.S. Dietary Reference Intake for an adequate intake of vitamin K for adult men is 120 micrograms/day and for adult women 90 micrograms/day [20].

### Habitual dietary intake

Additional information on dietary intake was obtained using a semi quantitative food frequency questionnaire (FFQ) that inquired about intake of 177 food items. For each item, the frequency was recorded in times per day, week, or month. The number of servings per frequency was expressed in natural units or household measures. The questionnaire was self-administered and filled out at home. Before participation in this study, all patients were carefully instructed by a trained researcher on how to complete the three day dietary diaries. In addition, similar written instructions were provided at the first page of the diary. The FFQs were checked for completeness and inconsistent answers were verified with the patients. Additionally, all participants were instructed to collect a 24 hour urine sample according to a strict protocol. In addition, excretion of several urinary components was measured to infer dietary intake of additional dietary nutrients like sodium and potassium.

### Vitamin K status

Blood was drawn after an 8–12 h overnight fasting period in the morning after completion of the dietary diary. Vitamin K status was assessed by measuring dp-ucMGP. Before serum preparation, blood was kept for 20 min at room temperature. Plasma and serum were prepared by standard centrifugation and stored at −80°C until testing.

Circulating dp-ucMGP levels were determined in citrated plasma using a dual-antibody ELISA (VitaK BV Maastricht The Netherlands). In this assay, the capture antibody is directed against the non-phosphorylated MGP sequence 3–15 and the detection antibody against the ucMGP sequence 35–49 or the carboxylated MGP sequence 35–54, respectively, as described previously [2], [13]. Vitamin K insufficiency was defined as dp-ucMGP levels of >500 pmol/L [13].

### Additional parameters

Renal function was assessed by calculating 24 h urinary creatinine clearance (ml/min). Serum creatinine levels were determined using a modified version of the Jaffé method (MEGA AU 510, Merck Diagnostica, Darmstadt, Germany). Plasma and urinary concentrations of electrolytes and urea were measured using routine clinical laboratory methods, as were serum cholesterol, N-terminal-pro-brain natriuretic peptide (NT pro-BNP), high sensitivity CRP. Information on patients' health status, medical history and medication use was obtained from patient records. Questionnaires were used to obtain information on smoking behavior. Participants were classified as current smokers, former smokers, or never smokers. Body weight and height were measured while participants wore indoor clothing without shoes. Body Mass Index (BMI) was calculated as weight divided by height squared (kg/m^2^).

### Statistical analysis

Anthropometric, clinical and laboratory parameters were compared between subjects with normal versus poor vitamin K intake using the Mann-Whitney non-parametric test or chi square test where appropriate. Similar analyses were performed to compare the characteristics of patients with normal versus elevated plasma dp-ucMGP levels.

Subsequently, linear regression analysis was performed to identify independent determinants of plasma dp-ucMGP levels, according to a gradual modeling approach. We started with a model containing age, gender, and renal function. Subsequently, parameters that significantly differed between subjects with normal versus poor vitamin K status were subsequently put into the regression model. If significant, the parameter remained in the model; if not significant, the parameter was removed from the model. This strategy was chosen to avoid a too large number of degrees of freedom when all possible covariates were put into the model at once. Non-normally distributed variables were transformed to the natural log before entering into the regression model.

Data are presented as mean±standard deviation or median (interquartile range), depending on their distribution (normal or non-normal, respectively), or percentages. A two-sided p-value of <0.05 was considered statistically significant Statistical analyses were performed using SPSS 15.0 for Windows (SPSS Corp. Chicago, IL).

## Results

The study population consisted of 30 male and 30 female kidney transplant recipients, who were at median 75 [interquartile range 35–188] months after kidney transplantation. Creatinine clearance was 61 ml/min [42–77 ml/min]. A detailed overview of baseline parameters is presented in Table 1.

**Table 1:** Patient characteristics stratified for normal vs poor vitamin K intake.

	Total cohort(*n = 60*)	Normal vitamin Kintake (*n = 30*)	Poor vitamin Kintake (*n = 30*)	P*
Male gender, n (%)	30 (50%)	15 (50%)	15 (50%)	0.99
Age, years	56 (48–63)	55 (47–63)	57 (48–63)	0.84
BMI, kg/m^2^	24.1 (22.1–27.0)	23.6 (21.4–25.9)	25.1 (23.2–28.0)	0.03
SBP, mmHg	135±17	133 (123–147)	130 (120–142)	0.45
Current or previous smoking	31 (52%)	20 (67%)	11 (37%)	0.02
Presumed cause of ESRD, n (%)				
Glomerulonephritis/vasculitis	10 (17%)	5 (17%)	5 (17%)	0.99
Membranous glomerulopathy/FSGS	1 (2%)	1 (3%)	0 (0%)	0.33
Vascular disease/hypertension	5 (8%)	4 (13%)	1 (3%)	0.17
IgA nephropathy	5 (8%)	0 (0%)	5 (17%)	0.02
ADPKD and MCKD	17 (28%)	9 (30%)	8 (27%)	0.78
Diabetic kidney disease	1 (2%)	1 (3%)	0 (0%)	0.33
Urological origin	7 (12%)	1 (3%)	6 (20%)	0.05
Other/unknown	14 (23%)	9 (30%)	5 (17%)	0.23
Transplant characteristics				
Follow up since transplant, months	75 (35–188)	65 (34–179)	93 (33–199)	0.44
Type of last transplant				
Living donor	16 (26.7%)	10 (33%)	6 (20%)	0.25
Cadaveric donor	44 (73.3%)	20 (67%)	24 (80%)	0.25
Transplant received				
Kidney only	57 (95%)	28 (93%)	29 (97%)	0.57
Simultaneous liver-kidney transplant	2 (3.3%)	1 (3%)	1 (3%)	0.98
Simultaneous pancreas-kidney transplant	1 (1.7%)	1 (3%)	0 (0%)	0.33
Dialyis prior to kidney transplantation	55 (92%)	25 (83%)	30 (100%)	0.02
Dialysis vintage, months	31 (17–59)	28 (5–49)	45 (23–67)	0.05
Diabetes	8 (13%)	4 (13%)	4 (13%)	0.97
Hypertension	56 (93%)	30 (100%)	26 (87%)	0.04
Hypercholesterolemia	43 (72%)	20 (67%)	34 (77%)	0.40
Previous CVD	9 (15%)	5 (17%)	4 (13%)	0.73
Current medication use				
Immunosuppressive drugs currently used				
Prednisolon	59 (98%)	30 (100%)	29 (97%)	0.33
Calcineurin inhibitor	34 (57%)	14 (47%)	20 (67%)	0.12
Ciclosporin	26 (76%)	10 (71%)	16 (80%)	0.12
Tacrolimus	8 (24%)	4 (29%)	4 (20%)	0.99
Mycophenolate mofetil	39 (65%)	21 (70%)	18 (60%)	0.43
Azathioprine	12 (20%)	7 (23%)	5 (17%)	0.53
mTOR inhibitor	2 (3%)	2 (7%)	0 (0%)	0.16
Statins	36 (60%)	17 (57%)	19 (63%)	0.61
Diuretics	16 (27%)	6 (20%)	10 (33%)	0.25
β-Blockers	40 (67%)	20 (67%)	20 (67%)	0.99
ACE inhibitors and/or AT1 blockers	28 (47%)	11 (37%)	16 (53%)	0.20
Calcium channel blockers	12 (20%)	6 (20%)	6 (20%)	0.99
Coumarin	6 (10%)	1 (3%)	5 (17%)	0.09
Aspirin	15 (25%)	9 (30%)	6 (20%)	0.38
Serum hsCRP, mg/dL	0.11 (0.04–0.28)	0.11 (0.04–0.15)	0.15 (0.05–0.49)	0.16
Serum creatinine, mg/dL	1.5 (1.2–1.8)	1.4 (1.3–1.8)	1.5 (1.2–1.9)	0.91
Serum uric acid, mg/dL	7.2 (5.8–8.4)	6.7 (5.7–8.0)	7.5 (6.1–8.6)	0.20
Serum total cholesterol, mg/dL	189 (170–228)	189 (174–224)	197 (162–232)	0.95
Serum NT pro-BNP, pg/mL	245 (113–719)	151 (106–476)	281 (130–863)	0.27
dp-ucMGP, pmol/L	753 (543–1091)	621 (481–927)	852 (620–1350)	0.04
24 hr urine sodium excretion, mmol/24 hr	130 (105–157)	115 (91–130)	147 (125–176)	0.001
24 hr urine urea nitrogen excretion, mg/24 hr	11 (9–12)	11 (9–13)	11 (9–12)	0.66
24 hr urine potassium excretion, mmol/24 hr	67 (55–88)	71 (60–92)	66 (53–84)	0.13
24 hr urine albumin excretion, mg/24 hr	94 (48–247)	63 (36–246)	120 (73–248)	0.11
24 hr urine creatinine cleareance, mL/min	61 (42–77)	61 (41–76)	62 (44–80)	0.69
Average daily dietary intake (U.S. Dietary Reference Intake for males [M], females [F] [35])				
Vitamin K1, µg/day	73.3 (36.0–153.2)	149.8 (110.1–270.3)	36.3 (22.7–59.5)	<0.001
Vitamin K2, µg/day	14.0 (5.3–25.3)	14.1 (4.7–28.9)	14.0 (5.8–22.7)	0.62
Total vitamin K, µg/day (M: 120, F: 90)	89.7 (53.2–174.7)	171.4 (137.8–282.9)	54.2 (45.2–77.3)	<0.001
Total energy, kJ/day (M: 9630, F: 7530)	8373 (7129–9404)	8487 (7229–9151)	7850 (6905–9566)	0.77
Total Kcal/day (M:2300, F: 1800)	1997 (1698–2236)	2017 (1723–2181)	1869 (1642–2279)	0.80
Total protein, g/day (10–35%^a^)	78.2 (67.8–86.1)	79.8 (69.1–91.3)	75.3 (65.5–82.0)	0.29
Vegetable proteins	28.7 (24.8–36.8)	29.3 (23.9–39.8)	28.2 (25.0–32.8)	0.14
Animal proteins	47.9 (40.5–54.0)	46.7 (40.5–56.4)	48.0 (40.4–52.4)	0.53
Carbohydrates, g/day (45–65%^a^)	183.9 (2.4–259.8)	215.7 (2.7–273.8)	156.6 (2.2–229.7)	0.19
Total fat, g/day (20–35%^a^)	73.0 (58.0–91.8)	73.0 (57.8–84.7)	73.8 (57.6–93.7)	0.71
Saturated fatty acids	27.0 (20.7–33.1)	25.4 (18.8–33.4)	28.1 (21.3–33.3)	0.46
Mono-unsaturated fatty acids	22.1 (18.2–29.4)	21.9 (19.0–29.0)	22.9 (17.7–30.3)	0.92
Poly-unsaturated fatty acids	14.2 (11.7–19.4)	13.9 (10.6–20.3)	15.3 (11.8–18.8)	0.83
Dietary fiber, g/day (M: 28, F: 22)	19.9 (0.04–26.6)	22.3 (0.4–29.2)	12.8 (0.02–21.9)	0.02
Potassium, mg/day (4700)	2986 (131–3881)	3548 (205–4017)	2393 (130–3401)	0.11
Alcohol, g/day (M<20, F<10)	10.8 (0–207.2)	3.2 (0–181.9)	21.7 (0.1–249.5)	0.05

Patient characteristics stratified for total vitamin K intake. Normal intake was defined as ≥120 µg/day (men) or ≥90 µg/day (women) [20].

* P value for comparison of subjects with normal vs poor vitamin K intake by Mann Whitney test. Nutritional goals apply to ages 51 years and older.

a Percentage of total energy intake.

Abbreviations: BMI, body mass index; SBP, systolic blood pressure; ADPKD, autosomal dominant polycystic kidney disease; MCKD, medullary cystic kidney disease; CVD, cardiovascular disease; mTOR, mammalian target of rapamycin; hsCRP, high-sensitivity C-reactive protein; NT-proBNP, N terminal-pro brain natriuretic peptide; MGP, matrix gla protein.

As shown in Table 1, total vitamin K intake was below the recommended level in 50% of all subjects, both in men and women. Lower vitamin K intake was associated with increased dp-ucMGP levels reflecting vascular vitamin K insufficiency. Patients with a low total vitamin K intake were more likely to have been on dialysis prior to transplantation and had a trend towards longer dialysis vintage (p = 0.05). Their diet contained more sodium, as reflected by an increased 24h-urinary sodium excretion (p = 0.001), and less dietary fiber (p = 0.02), with similar caloric intake between both groups. Dietary protein intake was positively associated with 24h-urinary urea excretion (r = 0.566, p<0.001), and dietary potassium intake was positively associated with 24h-potassium excretion (r = 0.468, p<0.001), suggesting that dietary diary information was representative of the actual intake as reflected by the 24h-urinary excretion. Analysis of the intake of specific dietary products (Table 2) from food frequency questionnaires revealed that patients with poor vitamin K intake tended to eat less green vegetables (p = 0.056), the most prominent dietary source of vitamin K1, as compared to subjects with normal vitamin K intake. Conversely, patients with poor vitamin K intake tended to use more milk (p = 0.06). A more detailed overview of dietary intake for both groups is provided in Table S1.

**Table 2:** Habitual dietary intake per vitamin K-containing food component assessed by food frequency questionnaires.

Dietary Component	Normal vitamin Kintake (*n = 30*)	Poor vitamin Kintake (*n = 30*)	p
Green Vegetables, g/d	39.8 (31.2–55.8)	32.6 (21.3–46.8)	0.06
Broccoli, cauliflower, g/d	14.9 (12.0–20.9)	11.6 (8.3–17.5)	0.05
Lettuce, spinach, endive, g/d	24.4 (16.4–36.8)	19.4 (6.9–30.5)	0.08
Cheese, g/d	27.4 (20.8–44.7)	27.2 (20.0–39.8)	0.47
Butter, g/d	34.6 (18.0–52.6)	25.6 (18.0–47.6)	0.47
Oil, g/d	0.62 (0.0–4.8)	0 (0.0–1.9)	0.19
Meat, g/d	32 (14.3–34.4)	26.9 (19–39)	0.42
Milk, g/d	146 (52–300)	259 (159–313)	0.06

Data are expressed as median (IQR).

Eighty percent of kidney transplant recipients had elevated dp-ucMGP levels, both in males (24/30) and in females (24/30). Vitamin K status was lower in patients on calcineurin inhibitors than in patients not using these drugs (dp-ucMGP levels 855 [590–1350] vs 616 [472–888] pmol/L, p = 0.006). On the contrary, dp-ucMGP levels were lower in subjects on azathioprine or cellcept (721 [504–917] vs 1073 [870–1733] pmol/L, p = 0.008) compared with patients not on these drugs. Separate analyses for both drugs revealed that patients on mycophenolate mofetil had lower dp-ucMGP levels as compared to those not on mycophenolate mofetil (591 [479–897] vs 917 [741–1412] pmol/L, p = 0.002), but the use of azathioprine did not influence dp-ucMGP levels (882 [703–1088] vs 726 [491–1091] pmol/L, p = 0.14). In patients using vitamin K antagonists (*n = 6*), dp-ucMGP levels were higher than in patients not on vitamin K antagonists (*n = 54*) with 725 pmol/L (516–923) vs 2079 pmol/L (1.658–2.272), p<0.001). Patients with elevated dp-ucMGP levels were more likely to have been on dialysis (96% vs 75%, p = 0.02) and tended to have had longer dialysis vintage (41 (17–61) vs 18 (1–30) months, p = 0.05) compared to patients with normal dp-ucMGP levels. Furthermore, patients with increased dp-ucMGP also had lower creatinine clearance (60 (45–75) vs 78 (52–83) ml/min, p = 0.04), higher levels of albuminuria (119 (62–304) vs 51 (30–75), p = 0.003, and tended to have higher NTproBNP levels (262 (118–825) vs 147 (70–355), p = 0.054) compared to patients with normal dp-ucMGP. All other variables (listed in Table 1) were not significantly different between vitamin K sufficient and insufficient patients.

Upon multivariate analysis creatinine clearance, coumarin use, body mass index, 24h-urinary sodium excretion (borderline) and high sensitivity CRP (borderline) were independent determinants of the vascular vitamin K status (Table 3). NT-proBNP levels and the type of immunosuppressive therapy were not significant determinants of vitamin K status in the multivariate model; their significance was lost when creatinine clearance was present in the model.

**Table 3:** Determinants of vascular vitamin K status (plasma dp-ucMGP).

	Standardized beta	P
**Model 1: Age, gender, creatinine clearance**		
Age	0.17	0.21
Gender	−0.17	0.22
Creatinine clearance	−0.43	0.001
**Model 2: Model 1 + coumarin use**		
Creatinine clearance	−0.32	0.002
Coumarin use (0 = no, 1 = yes)	0.52	<0.001
**Model 3: Model 2 + albuminuria**		
Creatinine clearance	−0.37	0.01
Coumarin use (0 = no, 1 = yes)	0.36	0.02
Albuminuria	0.04	0.79
**Model 4: Model 2 + pre-transplant dialysis**		
Creatinine clearance	−0.33	0.002
Coumarin use (0 = no, 1 = yes)	0.50	<0.001
Pre-transplant dialysis	0.18	0.07
**Model 5: Model 4 + high sensitivity CRP**		
Creatinine clearance	−0.37	0.001
Coumarin use (0 = no, 1 = yes)	0.32	0.009
Pre-transplant dialysis (0 = no, 1 = yes)	0.18	0.09
High sensitivity CRP	0.27	0.03
**Model 6: Model 5 + BMI**		
Creatinine clearance	−0.45	<0.001
Coumarin use (0 = no, 1 = yes)	0.36	0.001
Pre-transplant dialysis (0 = no, 1 = yes)	0.16	0.09
High sensitivity CRP	0.19	0.08
Body mass index	0.37	0.001
**Model 7: Model 6 + 24h-urinary sodium excretion**		
Creatinine clearance	−0.51	<0.001
Coumarin use (0 = no, 1 = yes)	0.31	0.005
Pre-transplant dialysis (0 = no, 1 = yes)	0.09	0.36
High sensitivity CRP	0.18	0.09
Body mass index	0.35	0.001
24h-urinary sodium excretion	0.22	0.05

Determinants of dp-ucMGP levels, reflecting vascular vitamin K status, analyzed by stepwise multivariate linear regression analysis. Abbreviations: dp-ucMGP, desphospho-uncarboxylated matrix gla protein; CRP, C-reactive protein; Non-normally distributed variables (creatinine clearance, albuminuria, high sensitivity CRP) were Ln-transformed before entering into the model.

## Discussion

The current study shows for the first time that both insufficient vitamin K intake and vascular vitamin K insufficiency (deduced from circulating dp-ucMGP levels) are very common in a population of stable kidney transplant recipients. Vitamin K deficiency in kidney transplant recipients has been reported previously by means of a coagulopathy responsive to vitamin K treatment in case series [21]. Mazzaferro et al recently argued that total MGP levels in kidney transplant recipients were close to normal [22]; however their study did not differentiate between carboxylated and uncarboxylated MGP which is important given the role for vitamin K in MGP carboxylation. When dp-ucMGP, an appropriate marker of vascular vitamin K status [2], [3], [23], was taken into account specifically in our study, we found clear indications of vascular vitamin K insufficiency in the majority of kidney transplant recipients.

Dietary recommendations for an adequate intake of vitamin K are based on the hepatic vitamin K1 requirement to for coagulation factor synthesis [20]. It does not account for vitamin K2 requirement to inhibit vascular calcification. The actual necessary amount of vitamin K intake based on the role of extra-hepatic vitamin K-dependent proteins is still unknown but studies suggest that dietary recommendations for vitamin K are too low to ensure full carboxylation of MGP [8]. Indeed, in our study even the subjects with adequate vitamin K intake according to U.S. guidelines [20] still had median dp-ucMGP levels above the recommended level of 500 pmol/L, suggesting vascular vitamin K insufficiency. This suggests that either kidney transplant recipients should be recommended to increase their dietary vitamin K intake beyond amounts recommended to the general population, or these patients should be supplemented with extra vitamin K.

Whether for vascular vitamin K status the intake of vitamin K2 is superior to vitamin K1 is uncertain, but the intake of vitamin K2 appears to be more important than vitamin K1 to prevent coronary heart disease [14]. Furthermore, in a recent pilot study in hemodialysis patients, a reduction of dp-ucMGP was dose-dependently achieved by treatment with vitamin K2 [11]. This can be mechanistically explained by the fact that the main transporters of vitamin K1 are triglyceride-rich lipoproteins that are retained by the liver and serve as a cofactor for proteins involved in coagulation. The vitamin accumulation and use in extrahepatic tissues such as the vascular wall, is low. Vitamin K2 on the other hand is transported not only by triglyceride-rich lipoproteins, but also by low density lipoproteins, the main carrier system to extrahepatic tissues [24]. In our study, subjects with poor vitamin K intake had increased dp-ucMGP levels (lower vascular vitamin K status) compared to those with normal vitamin K intake. On the other hand, dietary vitamin K intake (neither vitamin K1 nor K2 or total vitamin K) was not an independent determinant of dp-ucMGP levels upon multivariate analysis. This suggests that either other factors such as renal function, together with coumarin use as an iatrogenic factor, are more important determinants of vascular vitamin K status in this population. Dietary intake of vitamin K2 intake may also be an inappropriate reflection of the actual amount of vitamin K2 generated by intestinal micro-organisms. The composition of the intestinal flora, and importantly the presence of Bacteroides species, the main producers of vitamin K2 [25], is influenced by dietary factors including fibers [26]. The subjects with poor vitamin K intake in our population ate significantly less fiber than those with normal vitamin K intake; this may have affected intestinal vitamin K2 production.

The observation that vascular vitamin K insufficiency was more common than may be expected by vitamin K intake alone could be explained for a considerable part by the contribution of renal function impairment. Our data confirm the previously known relationship between renal function [22], [27] and dp-ucMGP levels and the influence of vitamin K antagonists [11]. Whether, as in hemodialysis patients [11], vitamin K2 supplementation may also reduce dp-ucMGP levels in kidney transplant recipients, especially those with compromised renal function, remains to be addressed in prospective trials. Our finding that vascular vitamin K status was associated with body mass index in multivariate analysis is in line with previous studies linking vitamin K status with parameters of glucose metabolism and atherosclerosis [28]. The borderline significant association between dp-ucMGP levels and 24h-urine sodium excretion suggests that high dietary sodium intake may negative affect vitamin K metabolism. Although vitamin K deficiency [1], [2] and high sodium intake [29] have both been associated with adverse cardiovascular outcomes, their possible interactions have not been addressed.

We found a trend towards an inverse relationship between vitamin K status and low-grade inflammation. Cell culture studies have shown anti-inflammatory effects of vitamin K in lipopolysaccharide-treated fibroblasts through inhibition of interleukin-6 [30]. In animals, a vitamin K-deficient diet enhanced the expression of inflammatory genes, which was reversed by vitamin K1-supplemented diets; furthermore the supplemented diet suppressed the inflammatory response induced by lipopolysaccharide [31]. Recently, both plasma vitamin K status and intake were inversely related to inflammatory markers in a human general population cohort [32].

The reduced vascular vitamin K status in patients on calcineurin inhibitors is in line with the increased risk of cardiovascular complications in patients on these drugs, particularly ciclosporin [33]. On the other hand, in our multivariate analysis of determinants of vitamin K status, none of the immunosuppressive drugs remained in the model after co-adjustment for creatinine clearance, suggesting that the differences in vitamin K status can be explained by differences in creatinine clearance. A recent report suggested that MGP levels were higher in patients on mTOR inhibitors [34]; unfortunately our cohort contained only two patients using this class of drugs.

Our study has the limitation of being a relatively small single center study in Caucasian patients only, so the generalizability of our findings will require support by studies in other populations. The limited sample size may have influenced the results of multivariate regression analysis, e.g. regarding the role of immunosuppressive regimens as determinants of vitamin K status. Furthermore, although dp-ucMGP levels have been associated with cardiovascular morbidity and mortality in the chronic kidney disease population [1], [2], these associations have not yet been established for the kidney transplant population.

In conclusion, we found that elevated dp-ucMGP levels, reflecting vascular vitamin K insufficiency, is common in kidney transplant recipients. Poor vitamin K intake is common in renal transplant recipients, and our data suggest that other factors including renal function may contribute to poor vascular vitamin K status as well. Correction of vitamin K status might be clinically relevant, given the known associations of vascular vitamin K deficiency with cardiovascular outcomes. Whether this can be achieved by relatively simple dietary measures in kidney transplant recipients should be addressed in future prospective studies.

## Supporting Information

Table S1 Detailed overview of dietary components for patients with normal as compared to poor vitamin K intake.
(DOC)
