# Supplementary material for: Vitamin K Intake and Plasma Desphospho-Uncarboxylated Matrix Gla-Protein Levels in Kidney Transplant Recipients
Source: PLoS One. 2012 Oct 31;7(10):e47991. doi: 10.1371/journal.pone.0047991 (PMC3485347; doi:10.1371/journal.pone.0047991)
Supplement: Table S1 — Detailed overview of dietary components for patients with normal as compared to poor vitamin K intake. (DOC) [file pone.0047991.s001.doc]

**Supplementary Table S1. Detailed overview of dietary components for patients with normal as compared to poor vitamin K intake**

| **Dietary Component** |  | **Normal vitamin K**  **intake (*n=30*)** |  | **Poor vitamin K**  **intake (*n=30*)** |  | **P*** |
| --- | --- | --- | --- | --- | --- | --- |
| **Vegetables** |  | 73.4 [60.0-98.1] |  | 69.7 [44.6-80.6] |  | 0.29 |
| Green vegetables |  | 39.8 [31.2-55.8] |  | 32.6 [21.3-46.8] |  | 0.056 |
| Broccoli, cauliflower, g/d |  | 14.9 [12.0-20.9] |  | 11.6 [8.3-17.5] |  | 0.05 |
| Lettuce, spinach, endive, g/d |  | 24.4 [16.4-36.8] |  | 19.4 [6.9-30.5] |  | 0.08 |
| Legumes, g/d |  | 31.9 [23.9-45.1] |  | 35.0 [22.2-37.3] |  | 0.88 |
| **Cheese** |  | 27.4 [20.8-44.7] |  | 27.2 [20.0-39.8] |  | 0.47 |
| 20+/30+ cheese, g/d |  | 0 [0-6.8] |  | 0 [0-12.5] |  | 0.92 |
| 40+ cheese, g/d |  | 0 [0-0] |  | 0 [0-0] |  | 0.40 |
| Regular cheese (48+), g/d |  | 8.3 [0.30] |  | 6.6 [0-20] |  | 0.41 |
| **Butter** |  | 34.6 [18.0-52.6] |  | 25.6 [18.0-47.6] |  | 0.47 |
| Margarine, g/d |  | 11.0 [0.0-29.6] |  | 18.0 [0.0-33.1] |  | 0.64 |
| Diet margarine, g/d |  | 18.2 [0-26.6] |  | 0 [0-20] |  | 0.04 |
| **Oil** |  | 0.62 [0.0-4.8] |  | 0 [0.0-1.9] |  | 0.19 |
| Olive oil, groundnut oil, g/d |  | 0 [0-1.23] |  | 0 [0-0] |  | 0.63 |
| Sunflower oil, others, g/d |  | 0 [0-0] |  | 0 [0-0] |  | 0.41 |
| **Meat** |  | 32 [14.3-34.4] |  | 26.9 [19-39] |  | 0.42 |
| Beef, g/d |  | 10.6 [0-12.7] |  | 7.65 [0-18.2] |  | 0.89 |
| Minced meat, g/d |  | 11.3 [6.29-13.45] |  | 9.9 [0-14.17] |  | 0.49 |
| Luncheon meat, g/d |  | 0 [0-2.29] |  | 0 [0-4.55] |  | 0.49 |
| **Milk products** |  | 146 [52-300] |  | 259 [159-313] |  | 0.06 |
| Whole milk, g/d |  | 0 [0-0] |  | 0 [0-0] |  | 0.55 |
| Semi-skimmed milk, g/d |  | 0 [0-74.4] |  | 107 [21.4-171.4] |  | 0.003 |
| Buttermilk, g/d |  | 0 [0-0] |  | 0 [0-21.4] |  | 0.35 |
| Whole yoghurt, g/d |  | 0 [0-0] |  | 0 [0-0] |  | 0.70 |
| Skimmed yoghurt, g/d |  | 0 [0-18.8] |  | 0 [0-44.7] |  | 0.28 |
